# Safety of Nicergoline as an Agent for Management of Cognitive Function Disorders

**DOI:** 10.1155/2014/610103

**Published:** 2014-08-28

**Authors:** Bernd Saletu, Amit Garg, Ahsan Shoeb

**Affiliations:** Section of Sleep Research and Pharmacopsychiatry, Department of Psychiatry, Medical University of Vienna, Waehringer Guertel, 1090 Vienna, Austria

## Abstract

Nicergoline is a semisynthetic ergot derivative and has a selective alpha-1A adrenergic receptor blocking property and also other additional mechanisms of actions, both in the brain and in the periphery. It is in clinical use for over three decades in over fifty countries for conditions such as cerebral infarction, acute and chronic peripheral circulation disorders, vascular dementia, and Alzheimer's disease and has been found to be beneficial in a variety of other conditions. However, concerns about its safety have been raised, especially after the European medicines agency's (EMEA's) restriction in the use of all ergot derivatives including nicergoline. But, most of the available literature and data suggest that the adverse events with nicergoline are mild and transient. Further, none of the available treatment options for cognitive disorders afford definitive resolution of symptoms. In this backdrop, we discuss the pharmacology of nicergoline with special emphasis on the safety of this compound, especially when used in patients suffering from cognitive function disorders.

## 1. Introduction

Cognitive function disorders (CFDs) are a group of disorders characterized by disruption in one or more of the cognitive domains. Diagnostic Statistical Manual of Mental Disorders, 5th edition (DSM-5), addresses CFDs as major neurocognitive disorder, which includes three groups of disorders, namely, delirium, dementia, and amnestic disorders [1]. All these three are characterized by an impairment in cognition (as in memory, language, or attention) [2].

Dementia has been estimated to affect about 6.1% of the world's population aged over 65 years. It has been predicted that there might be a considerable worldwide increase in the prevalence of dementia from 25 million in the year 2000 to 63 million in 2030 [3]. It is a clinical condition characterized by global impairment in cognitive function and is usually progressive impairment of cognitive functions. These functions include memory, judgement, reasoning, perception, and personality impairments. This leads to difficulties in daily life and to inappropriate behaviour. These symptoms are seen most often caused by Alzheimer's disease (AD) and ischemic damage to brain [4].

The management of AD is a multidisciplinary challenge, as currently only supportive treatment is available. Symptomatic pharmacological intervention is aimed at the treatment of the cognitive (cholinesterase inhibitors) and behavioural symptoms (psychotropic drugs) [3]. Amongst the cholinesterase inhibitors, donepezil, rivastigmine, galantamine, and tacrine are the drugs presently approved by the US Food and Drug Administration (FDA) [5]. Tacrine is no longer prescribed due to concerns of hepatotoxicity and unfavourable frequency of administration (4 times a day) [6]. Memantine is a useful psychotropic drug for AD, especially for moderate-to-severe disease but with little proven benefit in mild disease [7, 8]. However, each of these drugs has only modest efficacy for AD. Moreover, the controversy surrounding the usefulness of these antidementia medications is quite profound, especially as these agents slow the progression of cognitive decline but do not reverse the effects of the disease in AD [9].

The adverse events (AEs) reported with these drugs are mild and self-limiting. The anticholinesterases are associated to variable degrees with gastrointestinal effects, sleep disturbances, muscle cramps, weakness, bradycardia, and urinary incontinence, whereas memantine is associated with dizziness, confusion, headache, and incontinence [10].

For vascular dementia, treatment is largely symptomatic, and various agents reported to have modest efficacy include nicergoline, piracetam, oxiracetam, citicoline, pentoxifylline, propentofylline, aspirin, triflusal, and* Ginkgo biloba* [11].

**Figure 1:**
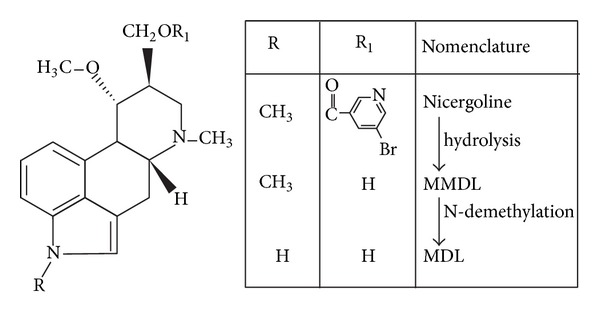
Structure of nicergoline and its metabolism into MMDL and MDL. The hydrolysis is catalysed by CYP2D6 [12].

Nicergoline, chemically 8-beta-(5-bromonicotinoylhydroxymethyl)-1,6-dimethyl-10alpha-metoxyergoline (Figure 1), is a semisynthetic ergot derivative which is registered in over fifty countries and in use for more than three decades for the treatment of cognitive, affective, and behavioural disorders in the elderly. Despite this status, concerns regarding the safety of nicergoline have been raised, as it is an ergot derivative [4].

The European medicines agency's (EMEA's) Committee for Medicinal Products for Human Use (CHMP) in its recommendations suggested that ergot containing medicines, including nicergoline, should no longer be used to treat conditions due to vascular aetiology (such as peripheral artery disease, Raynaud's syndrome, and retinopathies of vascular origin), in the prevention of migraine headaches or in the symptomatic treatment of venolymphatic insufficiency. This recommendation has been supported by the EMEA as ergot derivatives have a high likelihood of causing serious adverse events (SAEs) such as fibrosis and ergotism [12, 13]. However, in the same recommendations, it is suggested that healthcare professionals should stop prescribing ergot derivatives for the symptomatic treatment of neurosensorial impairment in the elderly, notably excluding Alzheimer's disease and other dementias.

In the backdrop of these recommendations, and keeping in mind the dearth of efficient drugs for CFD, we plan to explore the safety of nicergoline for this indication. In this paper we have reviewed the pharmacology of nicergoline with a special emphasis on the various AEs reported when being used as an agent to treat CFDs of various origins.

## 2. Pharmacology

Similar to other ergot derivatives, nicergoline is a potent and selective alpha-1A adrenergic receptor antagonist [14]. In addition, nicergoline is also reported to have the following actions:

- enhancement of catecholaminergic (noradrenaline and dopamine) turnover [15];
- stimulation of cholinergic neurotransmission, both by increased acetylcholine release from cholinergic nerve terminals and by selective enhancement of choline acetyltransferase enzyme [3];
- stimulation of phosphoinositide pathway and protein kinase C (PKC) *α* and *β* translocation [3];
- promotion of cerebral metabolic activity resulting in increased metabolism of oxygen and glucose [16];
- neuroprotective and antioxidant properties [17];
- antithrombotic activity via inhibition of platelet phospholipase, thus interfering with platelet aggregation [18, 19].

## 3. Rationale for Using Nicergoline in CFD

Nicergoline was initially considered to be useful in cerebrovascular disorders because of its vasoactive properties. Improvement in cerebral circulation was observed in patients with cerebrovascular disease when nicergoline was administered [20]. With the emergence of new information, a more complex pharmacological profile was proposed for nicergoline, which led to its consideration for the treatment of AD and other forms of dementia. Fioravanti and Flicker suggested that nicergoline may enhance dopaminergic and noradrenergic turnover in some areas of the brain and stimulates the phosphoinositide pathway which has been documented to be specifically impaired in AD. There is also an increase in the availability of acetylcholine by increasing its release and by inhibiting its degradation [4]. Nicergoline also increases phosphoinositide-PKC translocation, which combats beta-amyloid deposition and retards the reduction in nerve-growth factor (NGF), which may help in preventing cholinergic neuron loss [3, 4, 21]. It is also known to enhance intracellular levels of transforming-growth factor-*β* and glial-derived neurotrophic factor in astrocytes, two trophic factors that are known to protect neurons against *β*-amyloid toxicity [22, 23]. Further, nicergoline is postulated to improve vigilance and information processing at the neurophysiological level, leading to clinical improvement in degenerative and vascular dementia [24].

A definite diagnosis of advanced AD was determined by a clinical history of gradual decline in cognitive function and pathological finding of abundant NFT (neurofibrillary tangles) and SP (senile plaques) in the neocortex and medial temporal lobe. Presently, the major goal of research in AD is prevention of the disease, which entails early detection before the onset of symptoms. The concept of mild cognitive impairment (MCI), earliest clinically detectable phase of dementia and AD, has gained importance. Mild cognitive impairment is a clinical diagnosis and neuropathologic findings are just beginning to be defined. It appears now that most amnestic mild cognitive impairment (aMCI) patients are on a pathway toward AD [25].

The antiamnestic action of nicergoline was studied on passive avoidance response models such as amnesia induced by maximal electroshock in mice, scopolamine-induced amnesia in mice, and amnesia by paradoxical sleep deprivation in rats. Nicergoline demonstrates well-expressed antiamnestic effect in all these models and effect was equal to piracetam, meclofenoxate, pyritinol, deanol, and phenazepam [26]. Similar antiamnestic response to nicergoline was also obtained in the study conducted by Rakhmankulova and Voronina, displaying an equal-efficacy in comparison with piracetam and meclofenoxate [27]. According to a cochrane review, nicergoline has demonstrated a positive effect on cognition, behaviour, and clinical global impression in elderly patients with mild to moderate cognitive and behavioural impairment of various clinical origins (including chronic cerebrovascular disorders and AD) [4]. Considering the preclinical efficacy in antiamnestic activity along with neuroprotective properties of nicergoline, we cannot overlook its activity in aMCI. However, since almost all the clinical studies on nicergoline were reported long ago with no new information in the recent past, this drug has not been evaluated using current diagnostic categories. To further establish a disease modifying activity of this drug a long term double blind controlled clinical trials would be required.

## 4. Pharmacokinetics of Nicergoline

Nicergoline administered orally is rapidly and almost completely absorbed from the gut and is rapidly hydrolysed to an alcohol derivative, 1-methyl-10 alpha-methoxy-9,10-dihydrolysergol (MMDL), which is further N-demethylated to form 10 alpha-methoxy-9,10-dihydrolysergol (MDL). This reaction is catalysed to a major extent by CYP2D6 (Figure 1) [12, 24]. In addition, it undergoes metabolism by two minor pathways: (a) demethylation at position 1 of the ergoline nucleus; and (b) glucuronide conjugation of the free alcohols formed by the hydrolysis [28].

## 5. Uses of Nicergoline

Currently, where approved, nicergoline is being used as follows:

- to improve the apathy and affective disorders caused by cerebral infarction (such as reduced mental alertness, inattention, impairment of recent memory, hypobulia, and depression);
- in the treatment of acute and chronic peripheral circulation disorders (such as obliterative vascular disease of the limbs, Raynaud's syndrome, and other peripheral circulation dysfunction symptoms);
- in the treatment of vascular dementia, especially for the improvement in cognitive dysfunction and memory and to reduce the severity of this disease.

In addition, studies have reported the usefulness of nicergoline in posthemodialysis pruritus [29], tinnitus and vertigo [30], and ocular conditions such as arterial obstructions, venous thrombosis, diabetic retinopathies, senile macular degenerations, papilla ischaemic oedema, central serous chorioretinopathies, and glaucoma [31]. It has also displayed efficacy in the prevention of postoperative thrombophlebitis [32], management of balance disorders of central origin, Parkinson's disease, Leukoaraiosis, and benign prostatic hyperplasia [3]. A recent finding is that nicergoline increases serum substance P levels; this has been associated with reduction in the risk of aspiration pneumonia [33] and with improvement in dysphagia [34].

## 6. Concerns regarding Safety of Nicergoline

Although nicergoline has been approved for clinical use by regulatory authorities and extensively studied with respect to its efficacy and various modes of action, very limited data highlights the safety of nicergoline in subjects with cognitive decline. Long term safety of nicergoline is still best documented in comparison with placebo, and studies performed specially on patients with AD had very few subjects to be considered as absolute answers to the questions concerning the safety of use of nicergoline for this form of dementia. However, the authors of a cochrane review concluded that some evidence pointed towards an increased risk of adverse effects associated with nicergoline [4]. Further, the nicergoline cooperative study group (NCSG) reported that in comparison with placebo and ergoloid mesylates, nicergoline had a higher incidence of AEs [3]. Adverse events of nicergoline are mostly transient and minor, and are most often related to the central nervous system (CNS), the gastrointestinal system, and the cardiovascular system [3].

The NCSG reported the occurrence of CNS effects like hallucination, delusion, fatigue, and dysgeusia with nicergoline and not with either placebo or ergoloid mesylates [35]. Apart from this, nicergoline has been reported to cause diaphoresis, sleep disturbances, fainting, agitation, drowsiness, dizziness, insomnia, restlessness, flushing, and increased appetite [36, 37]. However, almost all of these latter reports are observed when nicergoline was compared with placebo. Even so, similarity in the frequency of these events between the two treatment groups was noted. All of these reactions were characterized as mild and transient did not impede continuation of treatment in all the studies reported.

A case report published in 2004 described a case of nicergoline-induced Prinzmetal angina in a 56-year-old hypertensive woman with history of right carotid endarterectomy; stoppage of nicergoline was associated with relief from the symptoms [38]. Apart from this case report, most of the cardiovascular events reported in association with nicergoline are minor and temporary and include temporary rise in BP [39], syncope [37, 40], bradycardia [40], and hypotension [40, 41]. Further, in a manner similar to the CNS adverse events, most of these cardiovascular events were seen when nicergoline was compared with placebo and did not require treatment withdrawal.

Nicergoline has been reported to cause minor gastrointestinal side effects such as heartburn [3], gastritis [37, 42], pyrosis, vomiting, diarrhoea, and abdominal pain [35]. All the other studies reported a similar frequency of gastrointestinal events with nicergoline and placebo-treated patients except the study conducted by NCSG.

Significant increase in serum uric acid levels was reported in trials; however, these patients did not require any intervention [4]. The development of gout in a previously hyperuricemic patient was reported by the NCSG in 1990, along with incidences of hyperuricemia in five patients, following therapy with nicergoline [4].

Various studies have reported other minor effects with nicergoline; these include hot flushes [37, 42], dizziness [41], inhibition of ejaculation [43], and interstitial nephritis [44]. While hot flushes can be due to the effects of nicergoline on blood vessels, inhibition of ejaculation is a known side-effect of alpha adrenergic blockers.

On the other hand, studies have reported that the tolerability of nicergoline at therapeutic doses is good [3, 41, 42, 45, 46]. In fact, no incidence of AEs was reported in almost 8 studies during the entire study duration [47, 48], though the possibility of poor methodology or data collection techniques cannot be ruled out in this study. Results from a recently published meta-analysis displayed no difference in the patient withdrawal rate between nicergoline and placebo group. Meta-analysis also displayed that the risk of any AEs was similar between nicergoline and placebo group, whereas risk of SAEs was slightly lower in nicergoline group; however, this difference was not statistically significant [48].

## 7. Risk of Fibrosis and Ergotism

There was a report in 1996 where four patients on nicergoline developed a syndrome of chronic pleural thickening/effusion that slowly improved after drug withdrawal. However, also mentioned in the report was that similar adverse events have never been reported earlier with nicergoline [49].

Further, with respect to ergotism, it is interesting to note that despite being an ergot derivative, there have been no reports in the literature connecting nicergoline to ergotism. The reasons for the absence of significant literature linking of nicergoline with these two important SAEs that are noted with other ergot derivatives may be two-fold. (a) The long term safety of nicergoline has not been studied; or (b) nicergoline may be devoid of a significant risk of ergotism and of fibrosis. Also of note is that almost all the clinical trials on nicergoline were published over two decades ago.

A systemic review by Fioravanti et al. has found no reference to cases with fibrosis and/or ergotism with nicergoline use [48].

In this background, clinical trials exploring the long term safety of nicergoline are warranted, especially in elderly patients with cognitive dysfunction, since not too many effective drugs are available for this diverse condition, and remains an area of unmet need. Thus, it is not a wise prospect to lose a drug for an adverse event it might not be having.

## 8. Drug Interactions

Nicergoline is known to potentiate the cardiodepressant effects of propranolol [50]. Since nicergoline is metabolised to a major extent by CYP2D6 [12], there are possibilities for interaction with drugs which are substrates of CYP2D6 (such as carvedilol, S-metaprolol, amitriptyline, clomipramine, haloperidol, aripiprazole, chlorpheniramine, and ondansetron), inducers (dexamethasone and rifampin) and inhibitors (bupropion, fluoxetine, quinidine, citalopram, H1-antihistaminics, metoclopramide, etc.) of CYP2D6 [51]. Drug interactions could be particularly important with nicergoline when it is used in the treatment of senile dementia since it is a condition of the elderly in whom polypharmacy is often routinely used. Appropriate clinical trials are required to investigate any significant and clinically relevant drug interactions.

Nicergoline is a strong inhibitor of platelet aggregation and decreases blood viscosity. Patients concurrently taking anticoagulants or antiplatelet agents should be closely monitored throughout the duration of therapy with nicergoline [18].

## 9. Conclusions

Despite concerns being raised against safety of nicergoline, a review of reported studies suggests that the drug is a safe and efficacious option in the therapeutic management of patients with dementia. The drug has not been evaluated using currently available diagnostic categories or against other therapeutic agents for dementia such as cholinesterase inhibitors or antioxidants. Even though the safety evaluations show a good tolerability profile for nicergoline, the reported adverse effects observed are self-limiting and minor. Further, none of the available treatment options for dementia offer a definitive solution to this condition. With this background, we feel that it is not wise to blindly reject a novel and useful drug such as nicergoline merely on the basis that it is an ergot derivative. We conclude that large and well-designed controlled studies using modern evaluation criteria and techniques are needed to demonstrate the efficacy and safety of nicergoline in dementia, including AD, in comparison with existing treatment options.
